# Adaptations of cytoarchitecture in human dilated cardiomyopathy

**DOI:** 10.1007/s12551-014-0146-2

**Published:** 2014-12-11

**Authors:** Marlene Pluess, Gregor Daeubler, Cristobal G. dos Remedios, Elisabeth Ehler

**Affiliations:** 1 Randall Division of Cell and Molecular Biophysics and Cardiovascular Division, British Heart Foundation Centre of Research Excellence, King’s College London, New Hunt’s House, Guy’s Campus, London, SE1 1UL UK; 2 Bosch Institute, Department of Anatomy, University of Sydney, Sydney, 2006 Australia

**Keywords:** Cytoskeleton, Intercalated disc, Dilated cardiomyopathy, Formin, M-band

## Abstract

Hypertrophic cardiomyopathy is characterised by a histological phenotype of myocyte disarray, but heart tissue samples from patients with dilated cardiomyopathy (DCM) often look comparatively similar to those from healthy individuals apart from conspicuous regions of fibrosis and necrosis. We have previously investigated subcellular alterations in the cytoarchitecture of mouse models of dilated cardiomyopathy and found that both the organisation and composition of the intercalated disc, i.e. the specialised type of cell–cell contact in the heart, is altered. There is also is a change in the composition of the M-band of the sarcomere due to an expression shift towards the more extensible embryonic heart (EH)-myomesin isoform. Analysis of human samples from the Sydney Human Heart Tissue Bank have revealed similar structural findings and also provided evidence for a dramatic change in overall cardiomyocyte size control, which has also been seen in the mouse. Together these changes in cytoarchitecture probably contribute to the decreased functional output that is seen in DCM.

In human heart disease the heart changes in one of two ways, either hypertrophy or dilation (Seidman and Seidman 2001). In hypertrophic cardiomyopathy (HCM), the walls of the ventricle thicken, which leads to a restricted chamber volume. HCM is usually identified histologically by a marked pattern of myocyte disarray in addition to fibrosis. HCM is additionally characterised by the re-expression of a set of marker genes that are usually not detectable in the postnatal heart, including those for atrial natriuretic peptide (ANP) and brain natriuretic peptide (BNP) or of the embryonic isoforms of, for example, contractile proteins such as myosin and actin (MacLellan and Schneider 2000; Copeland et al. 2010).

In dilated cardiomyopathy (DCM), the heart balloons and the chamber volume increases (Seidman and Seidman 2001). This condition can be accompanied by a thinning of the ventricular wall. The heart tissue of DCM patients can have an apparently normal appearance at the histological level, with nicely arranged strands of myocytes running in parallel. While there are areas of necrosis and fibrosis, often the majority of the tissue looks comparatively normal, thereby providing no good explanation for the dramatic failure in function that is seen at the physiological level. To date, only a very limited number of molecular markers specific for DCM have been defined.

## Alterations at the cellular level in mouse models of dilated cardiomyopathy

**Fig. 1:**
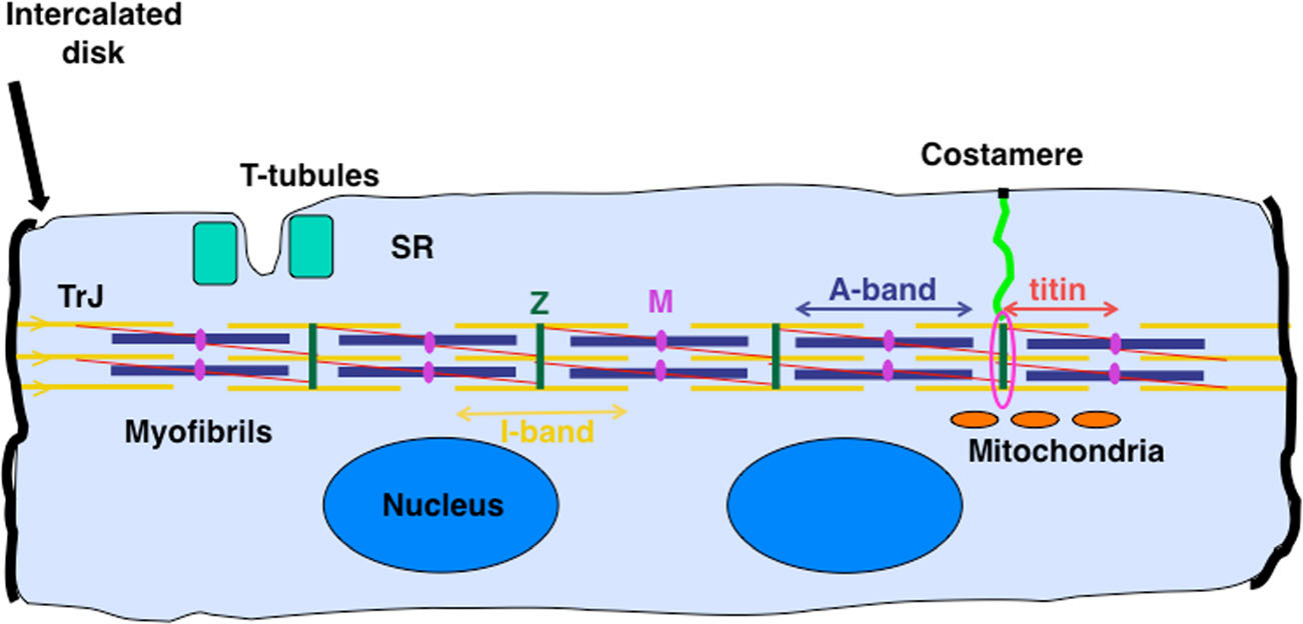
Schematic representation of an isolated adult cardiomyocyte. The regions of cell–cell contact, i.e. the intercalated discs, are situated at both ends of the cell. The cytoplasm is filled with myofibrils (just one shown here), which are arranged in a paracrystalline fashion and have sarcomeres which stretch between two Z-discs as the basic unit. Actin and its associated proteins (thin filaments, *yellow*) are found in the I-band, and myosin and its associated proteins (thick filaments, *dark blue*) are found in the A-band. One titin molecule (elastic filaments, *red*) stretches from the Z-disc to the M-band (shown in *magenta*), which is in the middle of each sarcomere and links the elastic filament system with the myosin filaments. The transitional junction (*TrJ*) is a proposed site for insertion of new sarcomeres in the region of the intercalated disc. At the sides the myofibrils are linked at Z-disc level with the surrounding extracellular matrix by structures called costameres

Our research group initially set out to explore potential alterations at the cellular level in DCM by employing mouse models of this disease (for a schematic representation of a single cardiomyocyte and its main structural components, see Fig. 1). In a first study, we used the MLP knockout mouse, which is the first genetically modified mouse model of DCM (Arber et al. 1997). We then went on to confirm our observations in a tropomodulin-overexpressing transgenic mouse (Sussman et al. 1998) and in a mouse that expressed a non-degradable version of the adherens junction protein beta-catenin (Hirschy et al. 2010). These studies focussed on the expression and localisation of different cytoskeletal proteins in the specialised type of cell–cell contacts found in the heart (intercalated discs) or in myofibrils. At the intercalated discs we saw a dramatic upregulation of proteins that anchor the sarcomeric actin filaments. Cytoplasmic components of the adherens junctions, such as beta-catenin and plakoglobin and their transmembrane counterpart, the catenins, were upregulated, contributing to an increased width of signal at the intercalated disc, as observed by immunohistochemistry and confocal microscopy (Ehler et al. 2001). The expression levels of desmosomal proteins were unchanged, but the levels of connexin-43, a component of the gap junctions involved in ion transfer between cardiomyocytes, were reduced. We also detected increased expression of nebulin-related anchoring protein (NRAP) and formin-homology-domain-containing protein 1 (FHOD1). Both are located at the intercalated disc, and the latter is a member of the formin family of regulators of actin filament formation (Ehler et al. 2001; Dwyer et al. 2014). The increased width at the intercalated disc seen by light microscopy following staining for actin-associated proteins was explained as an increased degree of membrane convolution, as has also been seen in ultrastructural analysis (Ehler et al. 2001).

Most sarcomeric proteins, such as alpha-actinin (a component of the Z-disc), myomesin (a component of the M-band) and thin and thick filament proteins [cardiac actin, sarcomeric myosin or MyBP-C (muscle protein myosin binding protein C)] do not alter their expression levels or their localisation patterns in DCM (Ehler et al. 2001). Schoenauer et al. (2011) observed a shift in isoform expression of myomesin towards the expression of EH (embryonic heart)-myomesin, which is usually predominant only in the embryonic heart muscle and in slow muscle. The myomesin molecule is made up almost exclusively of immunoglobulin and fibronectin type III domains (Agarkova et al. 2000), similar to other myosin binding proteins, such as MyBP-C and titin. EH-myomesin has an insertion of a domain in the middle of the myomesin molecule that appears to function in an analogous way to the PEVK domain of titin and displays more elastic properties in biophysical assays (Schoenauer et al. 2005). The composition of the M-band can be correlated to muscle type and developmental stage (Agarkova and Perriard 2005), and it has been speculated that this more extensible domain in EH-myomesin may be required for the function of myofibrils that have not perfectly aligned thick filaments, such as in the embryonic heart (Manasek 1968; Agarkova and Perriard 2005). In DCM, a similar switch in expression to a more extensible, embryonic isoform of a protein has been reported for titin (Makarenko et al. 2004), an elastic filament protein that spans all the way from the Z-disc to the M-band.

## Can similar changes be observed in samples from human patients with dilated cardiomyopathy?

Since inbred mouse strains are not necessarily representative of the heterogenous human population, the aim of our research group for several years has been to try to determine whether similar cytoskeletal alterations can also be seen in human hearts from DCM patients using samples from the Sydney Human Heart Tissue Bank that had always been stored in liquid nitrogen. Different types of DCM, as classified by the clinicians, were analysed in these studies, including idiopathic DCM (IDCM), familial DCM (FDCM) and DCM following a viral infection (VDCM).

**Fig. 2:**
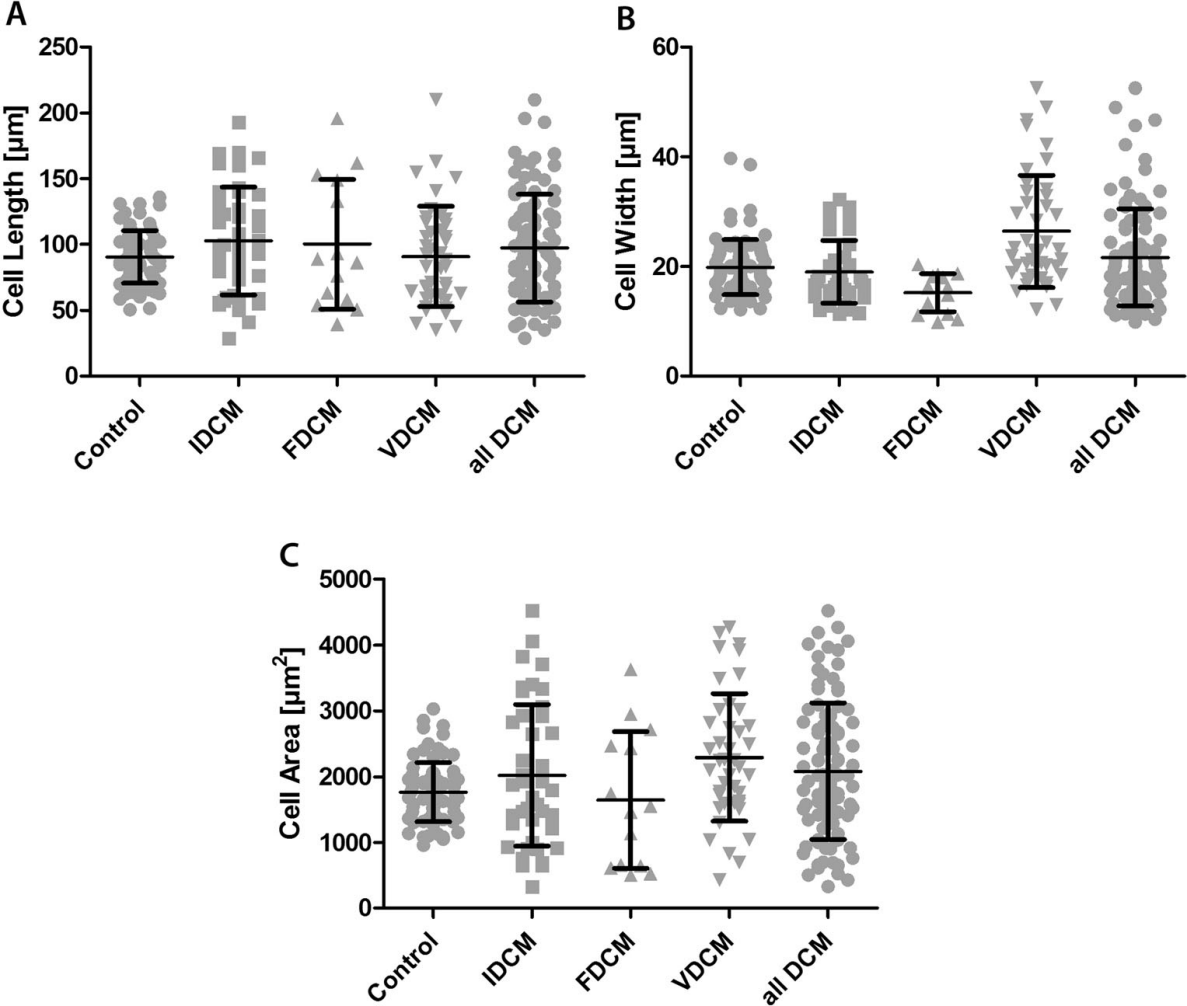
Analysis of size parameters of non-failing healthy cardiomyocytes and failing cardiomyocytes in dilated cardiomyopathy (DCM). Failing (DCM) cardiomyocytes show a larger range of measurements, indicating a departure from the usually narrow distribution of physiological size. Cell dimensions were obtained from confocal micrographs of longitudinally sections of cardiomyocytes stained with antibodies against beta-catenin and laminin to delineate the cell borders and myomesin. *Horizontal lines* Means, *error bars* standard deviations (SD), *grey symbols* individual measurements to show range and distribution. *IDCM* Idiopathic DCM, *FDCM* familial DCM, *VDCM* viral-caused DCM

Our initial analysis of different human DCM samples by immunohistochemistry followed by confocal microscopy revealed an increase in size distribution when the outlines of the cardiomyocytes were delineated by staining for beta-catenin and laminin. The traditional assumption is that DCM is caused by a general elongation of the cardiomyocytes that is accompanied by their slippage, leading to a thinning of the ventricular wall. Our measurements showed that while cardiomyocytes from non-failing hearts showed relatively little variability in size, diameter or length, cardiomyocytes from DCM patients of different etiologies were always characterised by an increased variation in these parameters (Fig. 2; Table 1). These findings mirror our initial studies on mouse models of DCM where we saw more variability in size—and not necessarily just elongation—of the cardiomyocytes (Leu et al. 2001; Hirschy et al. 2010). These observations suggest that the thinner wall of the DCM ventricle cannot always be explained solely by longer cells and that during the course of the disease cardiomyocytes lose some degree of size control. The signalling pathways which control cardiomyocyte growth are not very well understood at present (Földes et al. 2011).

**Table 1:** Length, width and area of non-failing control heart cardiomyocytes and cardiomyocytes in dilated cardiomyopathy

Measure^a^	Control (*n* = 80)	IDCM (*n* = 42)	FDCM (*n* = 14)	VDCM (*n* = 42)	all DCM (*n* = 98)
Maximum length (μm)	124.0	170.0	196.0	210.0	210.0
Minimum length (μm)	51.6	41.1	39.4	35.1	35.1
Range (μm)	72.5	128.9	156.6	174.9	174.9
Average length (μm)	90.4	102.7	100.2	91.0	93.2
Maximum width (μm)	39.7	30.6	20.4	52.5	52.5
Minimum width (μm)	12.3	11.4	9.9	12.2	9.9
Range (μm)	27.4	19.2	10.5	40.3	42.6
Average width [μm]	19.8	19.0	15.2	26.4	20.9
Maximum area (μm^2^)	2850.5	4060.0	3626.0	4268.4	4268.4
Minimum aera (μm^2^)	956.3	762.4	502.9	428.2	428.2
Range (μm^2^)	1894.2	3297.6	3123.1	3840.2	3840.2
Average area (μm^2^)	1756.3	2017.0	1643.3	2290.8	1937.4

*DCM * Dilated cardiomyopathy, *IDCM* idiopathic DCM, *FDCM* familial DCM, *VDCM* viral-caused DCM

^a^Non-failing and DCM heart sample sections were stained for laminin and beta-catenin to delineate the cell borders and myomesin for cardiomyocyte identification. The slides were co-stained with DAPI to indicate nuclei, and individual cells were measured at the nucleus level using LAS AF Lite software (Leica Microsystems, Wetzlar, Germany)

**Fig. 3:**
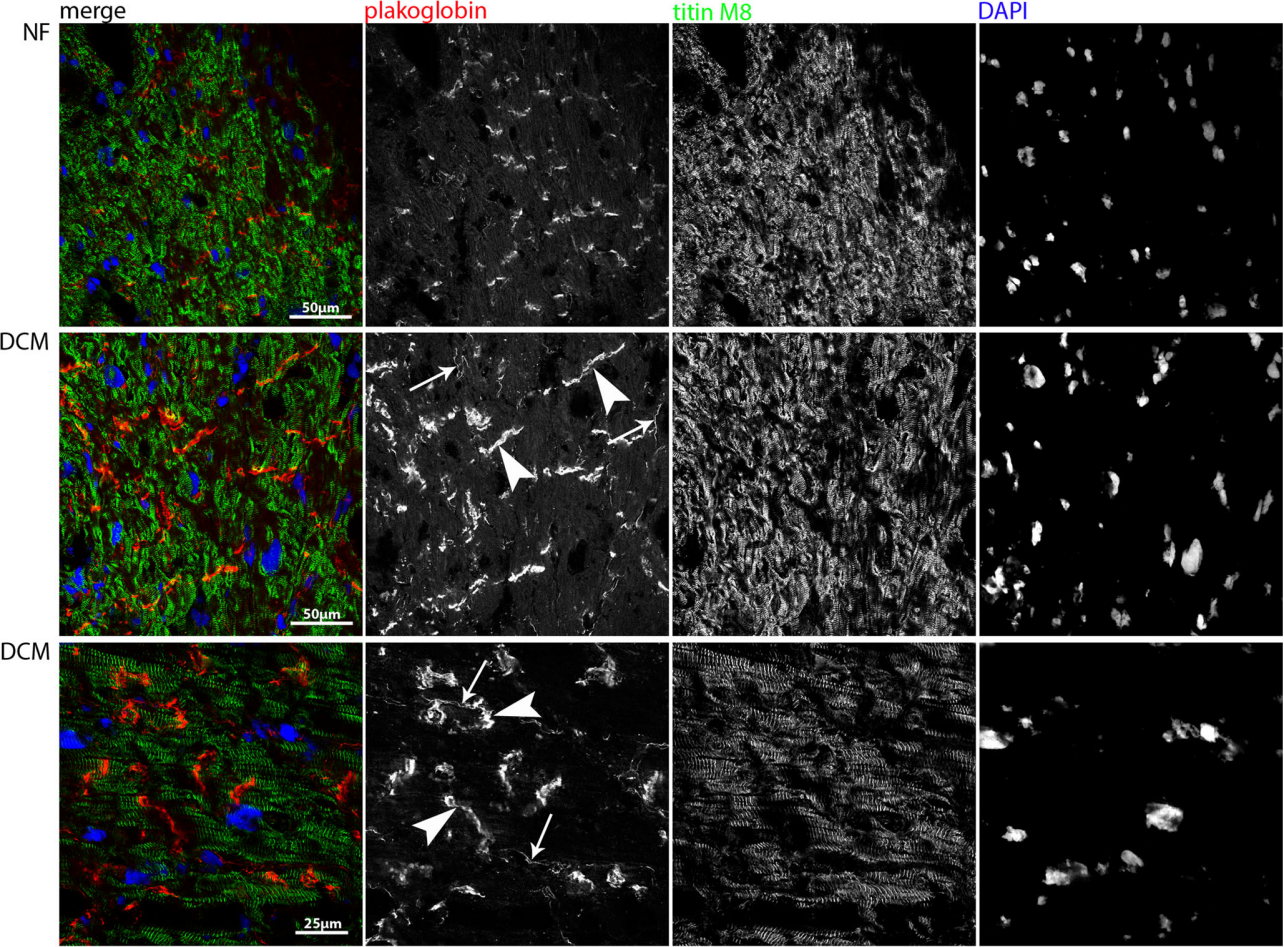
Increased signal for plakoglobin at the intercalated disc of DCM tissue samples. Confocal micrographs of cryosections from non-failing (healthy; *NF*) control and failing hearts (DCM). The intercalated discs were stained with monoclonal mouse antibodies against plakoglobin (*red* signal in overlay), the myofibrils were stained with polyclonal rabbit antibodies against the titin m8 epitope (*green* signal in overlay) and the nuclei were visualised with DAPI (*blue* signal in overlay). An increased width of signal for plakoglobin is apparent at the intercalated discs from tissue samples of different DCM patients compared to tissue from control (healthy) patients (*arrowheads*). A signal at the lateral edges of the cardiomyocytes can be occasionally detected in DCM (*arrow*)

When intercalated disc composition was analysed, we again observed increased protein levels for plakoglobin at the intercalated discs of the failing (DCM) patients compared to the non-failing controls (Fig. 3). This protein is found in adherens junctions, which anchor actin filaments, and in desmosomes, which anchor intermediate filaments. The concept that these two types of cell–cell contacts are clearly separated in cardiomyocytes has been recently challenged by the proposal of the “area composita”, demonstrated in the hearts of a variety of species, including humans (Franke et al. 2006). Interestingly, in samples from failing human hearts, a consistent downregulation of connexin-43 expression is not found: in some patients it is reduced, while in others the levels are comparable to those found in non-failing tissue (Pluess and Ehler, manuscript in preparation). At present it is unclear why failing (DCM) cardiomyocytes in humans respond differently to those in mice, but the effects of patient medication on connexin-43 expression cannot be excluded.

**Fig. 4:**
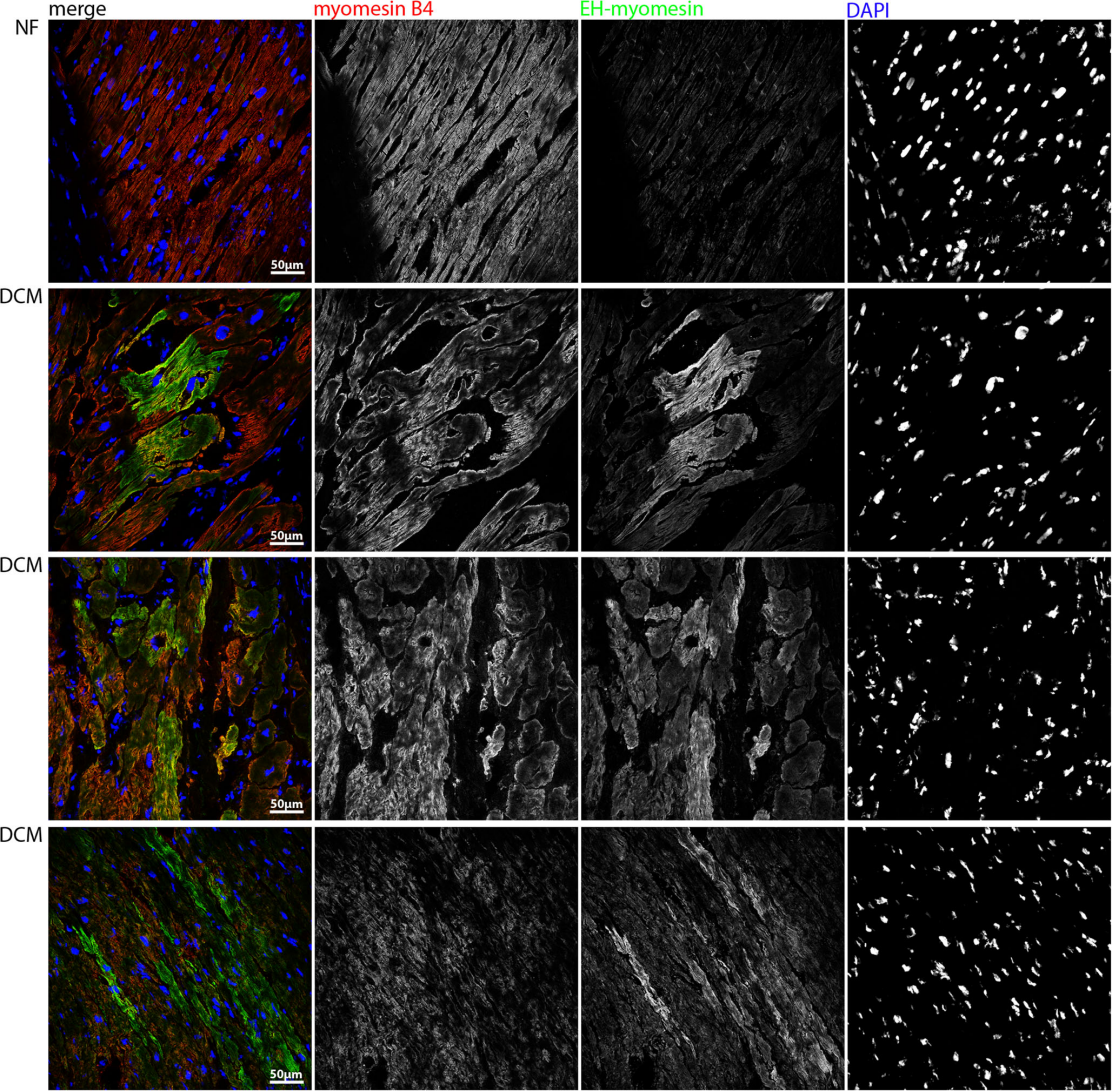
The more extensible, embryonic isoform of the M-band protein myomesin (*EH-myomesin*) is re-expressed in a cell-autonomous manner in DCM. Confocal micrographs of cryosections from non-failing healthy and DCM hearts. The M-bands were stained with monoclonal mouse antibodies against myomesin (*red* signal in overlay), the EH-myomesin isoform was stained with polyclonal rabbit antibodies against the human EH domain (*green* signal in overlay) and the nuclei were visualised with DAPI (*blue* signal in overlay). While no *green* signal can be detected in the healthy human heart, individual cardiomyocytes show a re-expression of EH-myomesin

**Fig. 5:**
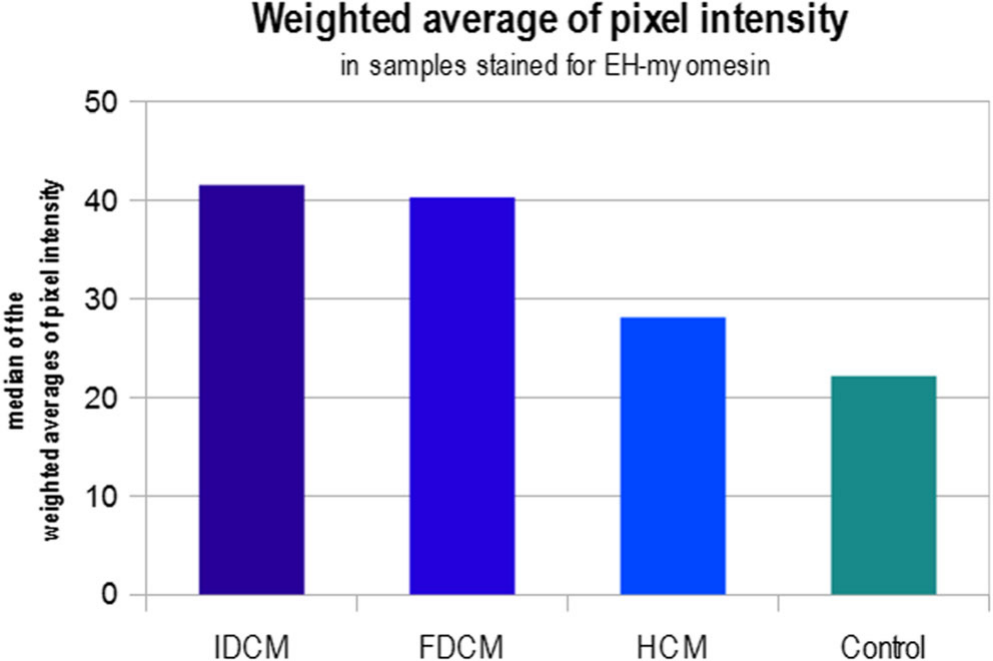
EH-myomesin expression is increased in IDCM and FDCM compared to HCM and healthy tissue samples. Quantification of pixel intensity from a tissue array containing 108 tissue spots for 54 individuals, stained with anti-human EH-myomesin antibodies and analysed by confocal microscopy. The values were grouped according to the underlying disease. A median of the weighted averages was determined for each subgroup: IDCM = 41.6; FDCM = 40.4; HCM = 28.1; non-failing healthy controls = 22.2

We found the general arrangement of sarcomeric proteins to be unaffected in both human and mouse DCM samples. As can be appreciated from the localisation pattern for titin or myomesin shown in Figs. 3 and 4, the cross-striations resemble those of the controls, i.e. there is not much evidence of dramatic myofibril disarray. Similar to the mouse, re-expression of EH-myomesin was also detected in human DCM hearts (Fig. 4). Upregulation of the expression of this embryonic isoform was seen in some cardiomyocytes and not in others. We made use of a tissue array with different cardiomyopathy samples and stained it for EH-myomesin expression. Analysis of the extent of expression revealed that there was a good correlation between high expression and IDCM and FDCM, but that lower expression of EH-myomesin occurred in HCM and non-failing hearts (Fig. 5). This makes EH-myomesin a potential marker for DCM. However, it must be noted that EH-myomesin is also expressed in the conduction system of the heart, particularly in the interventricular septum (Meysen et al. 2007); therefore it is important to note the location in the ventricle where the samples are taken.

**Fig. 6:**
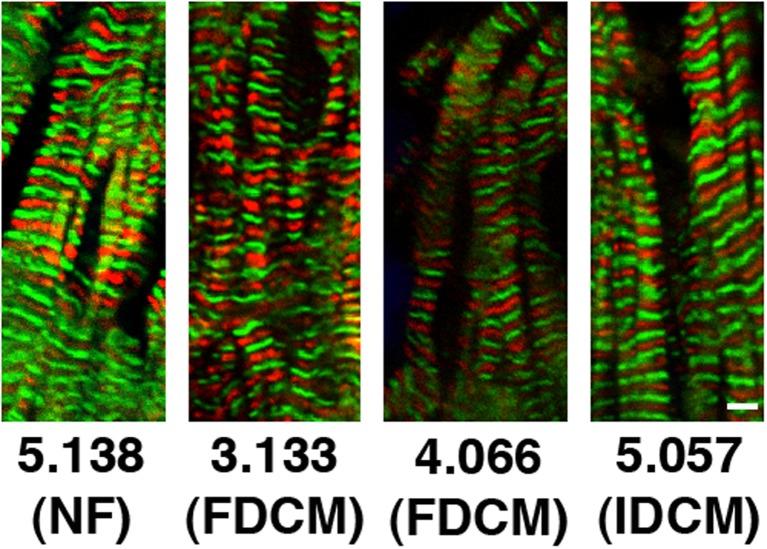
The titin T12 and m8 epitopes are stained with equal intensity in longitudinal sections of different DCM samples. Confocal micrographs of cryosections from non-failing (healthy; *NF*) and DCM hearts. The T12 epitope of titin in the Z-disc region was stained with monoclonal mouse antibodies (*red* signal). The m8 epitope of titin was stained with polyclonal rabbit antibodies (*green* signal) at the M-band. The Sydney Human Heart Tissue Bank patient codes are indicated. *Scale bar*: 2 μm

Approximately 25 % of cases of FDCM are caused by truncation of the titin gene (Herman et al. 2012). Next generation sequencing (NGS) of FDCM in the samples in the Sydney Human Heart Tissue Bank has commenced only recently, and no results have yet been published. Fürst et al. (1988) reported a monoclonal antibody (clone T12) against the N-terminal region of titin that recognises an epitope at the edge of the Z-disc, and Obermann et al. (1996) located a polyclonal antibody against the C-terminal titin m8 domain at the M-band. In all of the samples we have analysed to date, these two antibodies have revealed clear alterations of titin striations at apparently similar levels of intensity throughout the section (Fig. 6 and unpublished data). This result is surprising, since our expectation was fainter staining for the C-terminal epitope in some of the samples if they originated from patients with a truncated titin. However, since we currently have no NGS information on the genetic status of the samples used in our studies, we do not know whether these patients carry mutations in the titin gene that would lead to a truncated protein. Another complication of this research is that mutant titin may not necessarily be expressed at the expected ratio, which is 50 % since all the patients will be heterozygous. In the case of MyBP-C, truncating mutations can also cause HCM, and for this disease a haploinsufficiency mechanism has been proposed (Marston et al. 2009). Truncated MyBP-C has never been detected at the protein level, leading to the proposal that nonsense-mediated decay of the mRNA prevents its production (van Dijk et al. 2009) and may even prevent the translation in cases of point mutations in MyBP-C (Vignier et al. 2009). Mutations in other sarcomeric proteins, such as myosin, are not expressed at the expected 50 % ratio, but can be expressed at variable levels between patients and even between cardiomyocytes (Tripathi et al. 2011). The first report on a titin mutation causing DCM did show the existence of a truncated titin protein (Gerull et al. 2002). However, no biochemical analysis of titin has been carried out on the DCM samples available to us to date.

## Dilated cardiomyopathy: From cellular to functional phenotype?

**Fig. 7:**
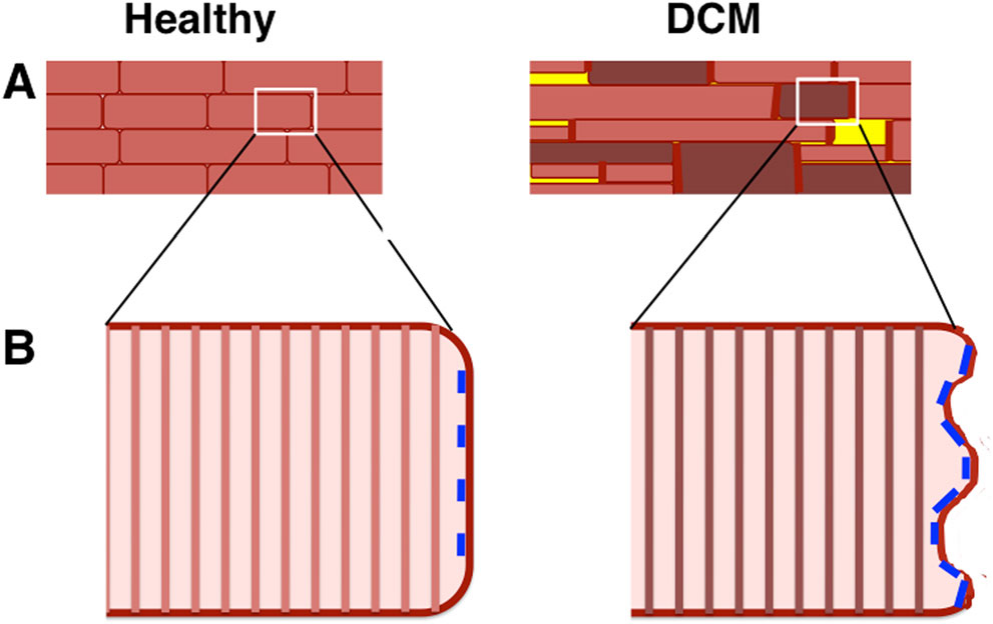
Schematic representations of the alterations at the level of the heart tissue (**a**) and the cardiomyocyte (**b**) in healthy and DCM hearts. **a** While the alignment of the cardiomyocytes is not greatly affected in DCM, there are patches of fibrosis (*yellow*) and a wider variation in cell size that lead to impaired coupling between cardiomyocytes. A subset of the cardiomyocytes (*darker colour*) re-express EH-myomesin. **b** The membrane at the intercalated discs is more convoluted in DCM cardiomyocytes. This is accompanied by an increased presence of actin anchoring adherens junctions (*blue*). The alignment of the myofibrils is unaffected, but there is an isoform shift in myomesin expression of the more extensible EH-myomesin at the M-band (*darker striations*)

What are the functional consequences of the changes that we observed in the DCM samples from mice and (wo)men? The higher variability in cell size will obviously affect the three-dimensional composition of the heart tissue and may be one of the explanations why we see these alterations in the cell–cell contact sites, i.e. the intercalated discs (for schematic representation of changes in DCM see Fig. 7). Suboptimal co-ordination between cell size and force per cell may necessitate an increase in actin anchorage to prevent tissue disintegration during contraction. It remains to be seen whether the formin FHOD1, detected at elevated levels at the intercalated discs of DCM in mice and humans (Dwyer et al. 2014), is responsible for the increased actin filament formation. The importance of the proper stoichiometry of expression of adherens junction protein resulting in heart failure has been reviewed elsewhere (Perriard et al. 2003; Vite and Radice 2014). NRAP is the first reported protein to be upregulated in the course of DCM (Ehler et al. 2001). Transgenic mice that overexpress this protein have dilated hearts and develop right ventricular dysfunction (Lu et al. 2011). The composition of the intercalated disc is important because it is the major site for the insertion of new sarcomeres during growth of the adult heart (Yoshida et al. 2010). In DCM hearts, this otherwise carefully regulated process leads to altered “step sizes” between the cells that no longer follow the “one step amounts to the length of one sarcomere” rule (Wilson et al. 2014). These irregularities are probably the basis for the increased degree of membrane convolution that is seen in the DCM heart and has also been reported for the aged heart (Forbes and Sperelakis 1985; Ehler et al. 2001). Other potential consequences of irregular tissue composition are fibrosis and changes in intercalated disc architecture that lead to an increased risk of arrhythmic events. Reduced expression of the gap junction protein connexin-43, as observed in all mouse models of DCM and a subset of the human patients, will impair intercellular communication and thus interfere with the efficient propagation of stimuli that produce contraction. In addition, lateral deposition of proteins such as plakoglobin and erroneous lateral gap junctions will contribute to a more arrhythmogenic phenotype (Severs et al. 2008).

The general composition of the myofibrils and arrangement of sarcomeres seems to be little affected in DCM, and myofibril disarray also appears to be minimal. There may be a failure in myofibril maintenance, which will eventually lead to shrinking of the cardiomyocytes or necrosis. For example, we correlated decreased expression levels of another formin, the muscle isoform of FHOD3, with human heart failure (Iskratsch et al. 2010). The composition of the M-band of the sarcomere is changed and reverts to a more embryonic phenotype. At present it is unclear whether the cell autonomous re-expression of EH-myomesin is beneficial. Schoenauer et al. (2011) reported that high EH-myomesin levels are correlated with a decrease in cardiac function in human samples. On the other hand, the initial reversion to embryonic phenotypes of myomesin and titin isoforms may be an adaptation to cope with increased mechanical stress. In the human samples we examined higher expression levels of EH-myomesin correlated with a longstanding disease and a low ejection fraction (Pluess and Ehler, manuscript in preparation).

The above observations point to the complimentary nature of the interactions of titin and myomesin and provide plausible explanations for the changes observed in DCM heart failure.

In summary, alterations that are seen at the level of tissue composition and in the arrangement of the cytoskeleton of cardiomyocytes appear to contribute to the functional changes that are seen at the level of physiology.

## References

[CR1] Agarkova I, Perriard JC (2005). The M-band: an elastic web that crosslinks thick filaments in the center of the sarcomere. Trends Cell Biol.

[CR2] Agarkova I, Auerbach D, Ehler E, Perriard J-C (2000). A novel marker for vertebrate embryonic heart, the EH-myomesin isoform. J Biol Chem.

[CR3] Arber S, Hunter JJ, Ross JJ, Hongo M, Sansig G, Borg J, Perriard J-C, Chien KR, Caroni P (1997). MLP-deficient mice exhibit a disruption of cardiac cytoarchitectural organization, dilated cardiomyopathy, and heart failure. Cell.

[CR4] Copeland O, Nowak KJ, Laing NG, Ravenscroft G, Messer AE, Bayliss CR, Marston SB (2010). Investigation of changes in skeletal muscle alpha-actin expression in normal and pathological human and mouse hearts. J Muscle Res Cell Motil.

[CR5] Dwyer J, Pluess M, Iskratsch T, Dos Remedios CG, Ehler E (2014). The formin FHOD1 in cardiomyocytes. Anat Rec (Hoboken).

[CR6] Ehler E, Horowits R, Zuppinger C, Price RL, Perriard E, Leu M, Caroni P, Sussman M, Eppenberger HM, Perriard JC (2001) Alterations at the intercalated disk associated with the absence of muscle LIM protein. J Cell Biol 153:763–772. *First detailed analysis of the dilated cardiomyopathy phenotype at the level of the cardiomyocyte using mouse models for this disease.* 10.1083/jcb.153.4.763 PMC2192386 11352937

[CR7] Földes G, Mioulane M, Wright JS, Liu AQ, Novak P, Merkely B, Gorelik J, Schneider MD, Ali NN, Harding SE (2011). Modulation of human embryonic stem cell-derived cardiomyocyte growth: a testbed for studying human cardiac hypertrophy?. J Mol Cell Cardiol.

[CR8] Forbes MS, Sperelakis N (1985). Intercalated discs of mammalian heart: a review of structure and function. Tissue Cell.

[CR9] Franke WW, Borrmann CM, Grund C, Pieperhoff S (2006) The area composita of adhering junctions connecting heart muscle cells of vertebrates. I. Molecular definition in intercalated disks of cardiomyocytes by immunoelectron microscopy of desmosomal proteins. Eur J Cell Biol 85:69–82. *The first in a series of publications that showed that the traditional segregation of desmosomes and adherens junctions is not followed in cardiomyocytes at a molecular basis and that the term “area composita” to define this type of cell-cell contact would be more appropriate.* 10.1016/j.ejcb.2005.11.003 16406610

[CR10] Fürst DO, Osborn M, Nave R, Weber K (1988). The organization of titin filaments in the half-sarcomere revealed by monoclonal antibodies in immunoelectron microscopy: a map of ten nonrepetitive epitopes starting at the Z line extends close to the M line. J Cell Biol.

[CR11] Gerull B, Gramlich M, Atherton J, McNabb M, Trombitas K, Sasse-Klaassen S, Seidman JG, Seidman C, Granzier H, Labeit S, Frenneaux M, Thierfelder L (2002). Mutations of TTN, encoding the giant muscle filament titin, cause familial dilated cardiomyopathy. Nat Genet.

[CR12] Herman DS, Lam L, Taylor MR, Wang L, Teekakirikul P, Christodoulou D, Conner L, DePalma SR, McDonough B, Sparks E, Teodorescu DL, Cirino AL, Banner NR, Pennell DJ, Graw S, Merlo M, Di Lenarda A, Sinagra G, Bos JM, Ackerman MJ, Mitchell RN, Murry CE, Lakdawala NK, Ho CY, Barton PJ, Cook SA, Mestroni L, Seidman JG, Seidman CE (2012). Truncations of titin causing dilated cardiomyopathy. N Engl J Med.

[CR13] Hirschy A, Croquelois A, Perriard E, Schoenauer R, Agarkova I, Hoerstrup SP, Taketo MM, Pedrazzini T, Perriard JC, Ehler E (2010). Stabilised beta-catenin in postnatal ventricular myocardium leads to dilated cardiomyopathy and premature death. Basic Res Cardiol.

[CR14] Iskratsch T, Lange S, Dwyer J, Kho AL, dos Remedios C, Ehler E (2010). Formin follows function: a muscle specific isoform of FHOD3 is regulated by CK2 phosphorylation and promotes myofibril maintenance. J Cell Biol.

[CR15] Leu M, Ehler E, Perriard JC (2001). Characterisation of postnatal growth of the murine heart. Anat Embryol (Berl).

[CR16] Lu S, Crawford GL, Dore J, Anderson SA, Despres D, Horowits R (2011). Cardiac-specific NRAP overexpression causes right ventricular dysfunction in mice. Exp Cell Res.

[CR17] MacLellan WR, Schneider MD (2000). Genetic dissection of cardiac growth control pathways. Annu Rev Physiol.

[CR18] Makarenko I, Opitz CA, Leake MC, Neagoe C, Kulke M, Gwathmey JK, del Monte F, Hajjar RJ, Linke WA (2004). Passive stiffness changes caused by upregulation of compliant titin isoforms in human dilated cardiomyopathy hearts. Circ Res.

[CR19] Manasek FJ (1968). Embryonic development of the heart. I. A light and electron microscopic study of myocardial development in the early chick embryo. J Morphol.

[CR20] Marston S, Copeland O, Jacques A, Livesey K, Tsang V, McKenna WJ, Jalilzadeh S, Carballo S, Redwood C, Watkins H (2009). Evidence from human myectomy samples that MYBPC3 mutations cause hypertrophic cardiomyopathy through haploinsufficiency. Circ Res.

[CR21] Meysen S, Marger L, Hewett KW, Jarry-Guichard T, Agarkova I, Chauvin JP, Perriard JC, Izumo S, Gourdie RG, Mangoni ME, Nargeot J, Gros D, Miquerol L (2007). Nkx2.5 cell-autonomous gene function is required for the postnatal formation of the peripheral ventricular conduction system. Dev Biol.

[CR22] Obermann WM, Gautel M, Steiner F, van der Ven PF, Weber K, Fürst DO (1996). The structure of the sarcomeric M band: localization of defined domains of myomesin, M-protein, and the 250-kD carboxy-terminal region of titin by immunoelectron microscopy. J Cell Biol.

[CR23] Perriard JC, Hirschy A, Ehler E (2003). Dilated cardiomyopathy: a disease of the intercalated disc?. Trends Cardiovasc Med.

[CR24] Schoenauer R, Bertoncini P, Machaidze G, Aebi U, Perriard JC, Hegner M, Agarkova I (2005). Myomesin is a molecular spring with adaptable elasticity. J Mol Biol.

[CR25] Schoenauer R, Emmert MY, Felley A, Ehler E, Brokopp C, Weber B, Nemir M, Faggian GG, Pedrazzini T, Falk V, Hoerstrup SP, Agarkova I (2011). EH-myomesin splice isoform is a novel marker for dilated cardiomyopathy. Basic Res Cardiol.

[CR26] Seidman JG, Seidman C (2001). The genetic basis for cardiomyopathy: from mutation identification to mechanistic paradigms. Cell.

[CR27] Severs NJ, Bruce AF, Dupont E, Rothery S (2008). Remodelling of gap junctions and connexin expression in diseased myocardium. Cardiovasc Res.

[CR28] Sussman MA, Welch S, Cambon N, Klevitsky R, Hewett TE, Price R, Witt SA, Kimball TR (1998). Myofibril degeneration caused by tropomodulin overexpression leads to dilated cardiomyopathy in juvenile mice. J Clin Invest.

[CR29] Tripathi S, Schultz I, Becker E, Montag J, Borchert B, Francino A, Navarro-Lopez F, Perrot A, Ozcelik C, Osterziel KJ, McKenna WJ, Brenner B, Kraft T (2011). Unequal allelic expression of wild-type and mutated beta-myosin in familial hypertrophic cardiomyopathy. Basic Res Cardiol.

[CR30] van Dijk SJ, Dooijes D, dos Remedios C, Michels M, Lamers JM, Winegrad S, Schlossarek S, Carrier L, ten Cate FJ, Stienen GJ, van der Velden J (2009). Cardiac myosin-binding protein C mutations and hypertrophic cardiomyopathy: haploinsufficiency, deranged phosphorylation, and cardiomyocyte dysfunction. Circulation.

[CR31] Vignier N, Schlossarek S, Fraysse B, Mearini G, Kramer E, Pointu H, Mougenot N, Guiard J, Reimer R, Hohenberg H, Schwartz K, Vernet M, Eschenhagen T, Carrier L (2009). Nonsense-mediated mRNA decay and ubiquitin-proteasome system regulate cardiac myosin-binding protein C mutant levels in cardiomyopathic mice. Circ Res.

[CR32] Vite A, Radice GL (2014). N-cadherin/catenin complex as a master regulator of intercalated disc function. Cell Commun Adhes.

[CR33] Wilson AJ, Schoenauer R, Ehler E, Agarkova I, Bennett PM (2014) Cardiomyocyte growth and sarcomerogenesis at the intercalated disc. Cell Mol Life Sci 71:165–181. *This manuscript focuses on the transitional junction, a kind of Z-disc light, which appears to be crucial in cardiomyocyte growth in the adult heart, by allowing the insertion of new sarcomeres and whose function appears to be compromised in dilated cardiomyopathy.* 10.1007/s00018-013-1374-5 PMC3889684 23708682

[CR34] Yoshida M, Sho E, Nanjo H, Takahashi M, Kobayashi M, Kawamura K, Honma M, Komatsu M, Sugita A, Yamauchi M, Hosoi T, Ito Y, Masuda H (2010). Weaving hypothesis of cardiomyocyte sarcomeres: discovery of periodic broadening and narrowing of intercalated disk during volume-load change. Am J Pathol.

